# Combination of contact ultrasound and infrared radiation for improving the quality and flavor of air-dried beef during hot air drying

**DOI:** 10.1016/j.ultsonch.2024.107047

**Published:** 2024-08-24

**Authors:** Jiahua Gao, Siyu Cheng, Xiaomei Sun, Yun Bai, Xiaobo Yu, Xianming Zeng, Songmei Hu, Minwei Zhang, Jianping Yue, Xinglian Xu, Minyi Han

**Affiliations:** a Key Laboratory of Meat Processing and Quality Control, Ministry of Education, Nanjing Agricultural University, Nanjing 210095, China; b Guangdong Testing Institute of Product Quality Supervision, Shunde 528300, China; c Emin County Xinda Tongchuang Bioengineering Co., Ltd., Tacheng 834600, China; d Wens Foodstuff Group Co., Ltd., Yunfu 527400, China

**Keywords:** Contact ultrasound, Infrared radiation, Air-dried beef, Quality, Flavor

## Abstract

Air-dried beef, a traditional dry fermented meat product in China, whose quality is largely influenced by processing conditions. In this study, contact ultrasound (CU) and infrared radiation (IR) were employed to enhance hot air drying (HAD), with an investigation into the mechanisms underlying improvements in quality and flavor. Samples subjected to CU and IR treatments during HAD (CU-IRD) demonstrated superior color (L* = 42.68, a* = 5.05, b* = −3.86) and tenderness (140.59 N) than HAD group, primarily attributed to reduced drying times and alterations in ultrastructure. Analyses utilizing SDS-PAGE and total volatile basic nitrogen (TVB-N) revealed that HAD and CU-HAD resulted in significant protein oxidation (197.85 mg TVB-N/kg and 202.23 mg TVB-N/kg, respectively), while IR treatments were associated with increased thermal degradation of proteins, producing lower molecular weight peptides. Compared with HAD group, the activities of certain lipases and proteases were enhanced by ultrasound and infrared treatments, leading to the release of greater amounts of free fatty acids and flavor amino acids. Furthermore, the thermal effects of infrared and the cavitation effects of ultrasound contributed to increased fat oxidation, amino acid Strecker degradation, and esterification reactions, thereby augmenting the diversity and concentration of volatile flavor compounds, including alkanes, ketones, aldehydes, and esters. These findings indicate that the synergistic application of CU and IR represents a promising strategy for enhancing the quality of air-dried beef.

## 1 Introduction

Air-dried beef jerky, a traditional fermented product in China, is known for its distinctive taste, flavor, and high nutritional value. To mitigate microbial growth and enhance storage stability, the jerky is dried to a moisture content of 10–50 g water per 100 g, achieving a water activity value of less than 0.850 [1]. However, traditional production methods, including natural air-drying and 35 °C hot air drying, are influenced by environmental conditions and equipment, resulting in extended processing times and diminished commercial value [2]. Furthermore, the degradation of essential qualities during processing, such as increased hardness, pronounced browning, and flavor loss, adversely affects consumer experience. To enhance the quality of the product, a more controllable and efficient processing method should be explored to substitute or improve the traditional techniques.

Infrared radiation (IR), as an independent heat source similar to microwave, does not require a medium for propagation. Radiant energy is capable of directly penetrating materials, inducing water molecules to vibrate at frequencies between 60,000 and 150,000 MHz, thereby generating heat and rapidly diminishing the thermal gradient of food within a short period [3]. The synergistic effect observed when hot air is combined with infrared or other heating methods results in enhanced heat transfer and dehydration efficiency, which is essential for alleviating surface hardening and color browning associated with prolonged hot air drying [4]. Flavor, a primary sensory characteristic of air-dried beef, significantly influences consumer preference and acceptance. Although there are no direct reports on how infrared radiation improves the flavor of meat products, insights can be drawn from several other aspects. Zheng et al [5] demonstrated that the combination of microwave and ultrasound improved the enzymatic hydrolysis of proteins, leading to an increased release of umami amino acids from bovine bone. It was speculated that microwaves induced structural changes in proteins, thereby exposing new enzymatic cleavage sites. Similarly, IR tends to facilitate the absorption of specific energy wavelengths by protein functional groups (C–H, C=O, N–H, etc.) in beef, potentially resulting in covalent bond breakage and protein denaturation, thus achieving effects comparable to those of microwaves [6]. Some aromatic compounds are prone to generate from some complex reactions during the baking process, such as Maillard reactions, lipid oxidation, or amino acid degradation. In comparison to conventional baking, Yu et al [7] found that IR enhanced the release of flavor compounds such as aldehydes, pyrazines, and fruity ketones from virgin rapeseed oils, attributed to higher heat fluxes and thermal expansion that produced a more pronounced baking effect than traditional method. Additionally, Bozkir et al [8] noted that IR reduced drying time, effectively contributing to the retention of volatile compounds in orange peels.

Ultrasound is an environmentally-friendly emerging technology that can shorten processing times and improve product quality, thereby offering significant benefits and promising prospects for application in the meat industry [9]. Low-frequency, high-power ultrasound (power ultrasound) is extensively utilized in food processing and extraction due to its destructive capabilities. The phenomena of cavitation, along with mechanical and thermal effects, accelerate mass transfer and decomposition in biological materials, resulting in modifications to texture, structure, and flavour [10]. Meat tenderness is improved primarily through high-frequency mechanical vibrations that periodically compress and stretch the material, leading to alterations in microstructure and increased fiber breakage. Additionally, the hydrolysis of structural proteins by lysosomes and proteases also play a critical role, facilitated by the release and enhanced activity of these hydrolytic factors due to ultrasound-induced cell rupture [11]. The cavitation mechanism induced by ultrasound initiates a series of micro-environmental changes, including localized high-temperature and high-pressure conditions, microjets, and agitation. These factors promote biochemical reactions such as the Maillard reaction, esterification, and Strecker degradation, significantly influencing flavor development in fermented foods [12]. Proteins and polyunsaturated fatty acids in beef are susceptible to interactions with free radicals generated by cavitation, leading to oxidation that produces additional volatile flavor compounds or flavor amino acids, particularly aldehydes, ketones, and umami and sweet amino acids [13].

In our previous research, the application of contact ultrasound (CU) and infrared radiation (IR) in hot air drying improved the heat transfer and moisture migration of air-dried beef [14], but the effect of them on the quality remain unclear. A hypothesis is that these two technologies may exhibit unique synergistic effects on quality and flavor development. Consequently, this study compared four different drying methods (with or without the application of CU and/or IR), aiming to elucidate the mechanisms of quality changes and flavor formation in air-dried beef during different processes.

## 2 Materials and methods

### 2.1 Sample preparation

The *longissimus dorsi* (LD) was obtained from a local commercial slaughterhouse (Yurun Meat Processing Company, Nanjing, China) within 24 h post-mortem, and the cattle were approximately 3 years old at slaughter. After removal of visible connective tissue and external fat, the meat was packaged in polythene bags and stored in a −18 °C freezer. Prior to use, the beef was thawed for 12 h at 4 °C and subsequently cut into strips measuring 1.5 cm × 1.5 cm × 4 cm for same-day use only. The initial moisture content on a wet basis (w. b.) was determined according to the method provided by Sánchez-Torres et al [15], was 74.82 ± 0.63 %.

### 2.2 Experimental equipment and drying procedures

**Fig. 1:**
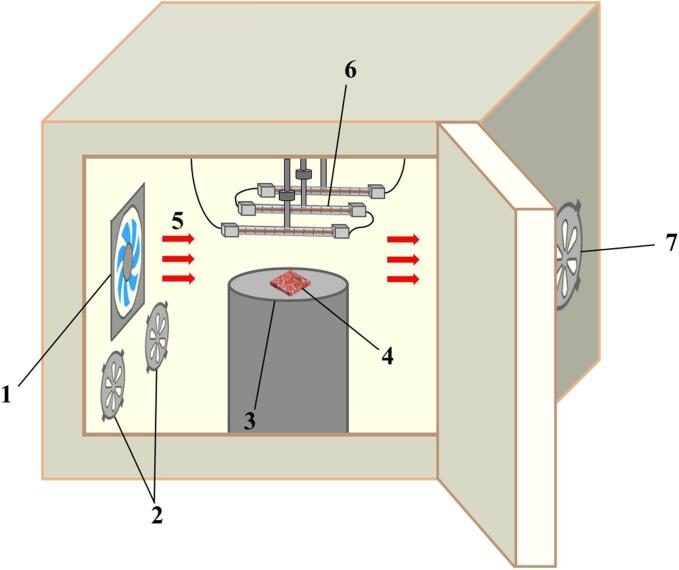
Schematic diagram of the combined dryer. 1. axial fan, 2. air inlet, 3. ultrasonic vibration plate, 4. beef samples, 5. airflow, 6. infrared lamp, 7. air outlet.

A hot air dryer equipped with a contact ultrasound system and an infrared emitter was used for processing (Fig. 1), as detailed in our previous study [14]. Three series-connected infrared tubes (IR74-115–300, Guangzhiyang Photoelectricity Technology Co., Ltd, China) were installed at the top of the dryer, with a total length of 300 mm and a heating length of 230 mm for each tube. The voltage applied across the tube assembly was set at 120 V (actual power 140 W), ensuring uniform radiation density and a controllable temperature of approximately 35 °C. Samples were placed on a 25 cm diameter, 20 kHz contact ultrasonic vibration plate (vibration mode 5 s on 5 s off) and the actual ultrasonic energy transmitted from the ultrasonic vibration plate was detected to be 12.6 W/dm^2^ [16].

Combinations of different drying conditions were selected for the experiment (Table 1), including with or without the use of contact ultrasound (CU) and/or infrared radiation (IR). For all drying conditions, the inlet and outlet temperatures of the hot air and the airflow rate were maintained at 35 °C and 4.5 m/s, respectively. The experiment was conducted until a weight loss of 50 % was achieved, corresponding to a target moisture content of 50 % (on a wet basis) at the conclusion of the drying process. According to our previous research [14], significant differences in both the time requirement and the internal equilibrium temperature of the samples were observed under various drying conditions (Table 1).

**Table 1:** Drying conditions used in this study.

Sample code	Contacting ultrasound (CU)	Infrared (IR)	Hot air	Drying time (h)	Internal temperature (℃)
HAD	−	−	+	9.5	30
CU-HAD	+	−	+	8.0	33
IRD	−	+	+	7.0	38
CU-IRD	+	+	+	6.0	38

Note: The “+” and “-” denote use and non-use, respectively. HAD: hot air drying, CU-HAD: hot air drying with CU, IRD: hot air drying with IR, CU-IRD: hot air drying with CU and IR.

### 2.3 Color measurements

The color of the sample was measured using a portable colorimeter (CR-400, Konica Minolta, Japan). Color values were represented by L* (lightness/darkness), a* (redness/greenness), and b* (yellowness/blueness). The instrument was calibrated on the Hunterlab color space system using a white reference tile (L* = 97.50, a* = −0.60 and b* = 2.30) and a D_65_ illuminant source before the measurements.

### 2.4 Shear force

The pieces with a size of 10 mm × 10 mm × 20 mm were precisely cut along the direction of the muscle fibers of air-dried beef. A digital muscle tenderizer (C-LM3B, Northeast Agricultural University, China) was used to measure the mechanical properties of the samples. During the test, the samples were sheared at a speed of 5 mm/s to obtain the corresponding shear force values, which were measured in N.

### 2.5 Ultrastructure

The ultrastructure of the samples was observed using a transmission electron microscope (HT7800, Hitachi, Japan) following the method of Luo et al [17]. The samples were cut into 5 mm × 5 mm × 5 mm cubes and pre-fixed using 2.5 % glutaraldehyde solution (4 °C) before being rinsed three times with 0.1 mol/L phosphate buffer (pH 7.0). Subsequently, they were post-fixed with 1 % osmium tetroxide (OsO_4_) for 2 h and rinsed repeatedly with phosphate buffer. The samples were then dehydrated through a graded series of ethanol solutions (30 %, 50 %, 70 %, 80 %) and absolute acetone. After dehydration was completed, the samples were embedded and then sectioned (70–90 nm) using an ultramicrotome (EM UC7, Leica, Germany). These sections were stained sequentially with lead citrate and uranyl acetate solutions before being observed under the transmission electron microscope.

### 2.6 Total volatile basic nitrogen (TVB-N)

Total volatile basic nitrogen (TVB-N) was determined according to the method described by Gharibzahedi [18]. An accurately weighed 2 g sample and 1 g of magnesium oxide were added to a distillation tube and distilled using a fully automated Kjeldahl nitrogen apparatus (Kjeltec 8400, Foss, Sweden). The distillate was collected in a container containing 2 % boric acid solution and a mixed indicator (0.1 % methyl red and 0.1 % bromocresol green in ethanol solution). Titration was performed using 0.1001 mol/L hydrochloric acid. The results were determined based on the volume of hydrochloric acid consumed and expressed as mg (TVB-N)/kg (meat).

### 2.7 Thiobarbituric acid reactive substances (TBARS)

Referring to the method of Luo et al [19], a 2.0 g homogenized sample was mixed with 10 mL of trichloroacetic acid (TCA) solution (17.5 % TCA, 0.1 % EDTA). The mixture was homogenized in an ice water bath at 10000 rpm using a high-speed homogenizer (PD500-TP, Prima, England). The homogenate was then centrifuged using a high-speed centrifuge (Avanti JXN-26, Beckman Coulter, USA) at 18000 × g and 4 °C for 5 min. Two milliliters of the supernatant was collected and mixed with 2 mL of thiobarbituric acid (TBA) solution (0.02 mol/L). This mixture was vortexed and then incubated in a water bath at 95 °C for 30 min. After cooling, the absorbance was measured at 532 nm using a microplate reader (Spark, Tecan, Switzerland). A standard curve was plotted using malondialdehyde (MDA) standards, and the degree of lipid oxidation in the sample was calculated based on this standard curve. The results were expressed as milligrams of MDA per kilogram of meat sample, i.e., mg MDA/kg meat.

### 2.8 Sodium dodecyl sulfate polyacrylamide gel electrophoresis (SDS-PAGE)

The aggregation and degradation of protein were detected according to the method of Kang [20]. A 1 g minced sample was mixed with 10 mL of 10 mmol/L K_2_HPO_4_/KH_2_PO_4_ buffer, homogenized by high-speed homogenizer (PD500-TP, Prima, England), centrifuged (10000×*g*, 4 °C, 10 min) using a high-speed freezing centrifuge (Avanti JXN-26, Beckman Coulter, USA), and the protein concentration of the supernatant was determined and adjusted to 1 mg/mL. The protein concentration of the final pellet was determined by the Biuret method [21] using bovine serum albumin (BSA) as the standard. The protein solution was then mixed with sample buffer (1 mol/L Tris-HCl, 50 % (v/v) glycerol, 10 % (w/v) SDS, and 1 % (w/v) bromophenol blue) containing either with or without 10 % (w/v) β-mercaptoethanol (β-ME) and heated at 95 °C for 5 min to denature the proteins. A volume of 10 µL of the sample and 5 µL of standard protein marker (M00624, GenScript, USA) were loaded onto a Bis-Tris precast gel with a gradient concentration of 4–20 %. Electrophoresis was performed using an electrophoresis apparatus (Mini-ProTEAN XCell, Bio-Rad, USA) at 80 V of concentration for 30 min, followed by separation at 120 V for 110 min. After electrophoresis, the gel was stained with Coomassie brilliant blue and destained with destaining solution. The gel was then scanned and analyzed on a gel imager (Universal Hood II, Bio-Rad, USA).

### 2.9 Free amino acids (FAAs)

The content of FAAs was analyzed according to the method of Zou et al [13]. In brief, 4 g of air-dried beef was homogenized with 20 mL of 3 % (w/v) sulfosalicylic acid (10000 rpm, 30 s) by high-speed homogenizer (PD500-TP, Prima, England). Subsequently, the homogenate was centrifuged at 4 °C and 18000 × g for 15 min using a high-speed centrifuge (Avanti JXN-26, Beckman Coulter, USA). The supernatant was mixed with hexane and the lower aqueous phase was filtered through a 0.22 µm filter membrane. The concentration of FAAs was determined using an automatic amino acid analyzer (L-8900, Hitachi, Tokyo).

### 2.10 Fatty acid composition

Lipid extraction and methyl esterification of air-dried beef were performed according to the method of Barido et al [22]. Briefly, 3 g of sample was mixed with 20 mL of chloroform/methanol solution (2:1, v/v) and homogenized by high-speed homogenizer (PD500-TP, Prima, England) at 10,000 r/min. The homogenized mixture was left to stand away from light (4 °C, 24 h) and then filtered through gauze. Four milliliters of saline (0.9 %, w/v) was added to the filtrate, mixed thoroughly, and centrifuged (3000 × g, 4 °C, 15 min) using a high-speed centrifuge (Avanti JXN-26, Beckman Coulter, USA). The organic solvent was removed from the lower organic phase by nitrogen blowing to obtain a pure fat. Five milliliters of 8 % (w/v) NaOH-CH_3_OH solution was added to the purified fat, and the mixture was heated in a water bath at 70 °C for 10 min. Subsequently, five milliliters of boron trifluoride-methanol solution (14 %, w/v) was added, and the above heating step was repeated. Seven milliliters of hexane and ten milliliters of saturated NaOH solution were added while the above solution was still hot, mixed thoroughly, and centrifuged (15000×*g*, 4 °C, 5 min) by high-speed centrifuge (Avanti JXN-26, Beckman Coulter, USA). The upper organic phase was filtered through a 0.22 µm organic filter membrane. The fatty acid composition of the filtrate was analyzed using an ultra-high-performance liquid chromatography system (UltiMate, Thermo, USA).

### 2.11 Electronic tongue

Following the method of Wang et al [23] with slight modifications, an electronic tongue (SA402B, Insent, Japan) equipped with five potentiometric chemical sensors (umami AAE, saltiness CT0, sourness CA0, bitterness C00, and astringency AE1) was used to assess the taste characteristics of different samples. Specifically, the procedure was as follows: A 5 g of sample was mixed with 100 mL of water (12000 rpm, 20 s) by high-speed homogenizer (PD500-TP, Prima, England) until homogeneous. The mixture was then centrifuged at 13000×*g* for 15 min using a high-speed centrifuge (Avanti JXN-26, Beckman Coulter, USA). The supernatant obtained after centrifugation was filtered through a 0.22 µm aqueous filter membrane and used for taste measurement.

### 2.12 Volatile flavor compounds

Volatile flavor compounds were extracted from the air-dried beef using headspace solid-phase microextraction (HS-SPME) and analyzed by gas chromatography-mass spectrometry (GC–MS) (TSQ9000, Thermo Fisher, USA) using the method of Xu et al [24]. A 2 g sample was placed in a 20 mL headspace vial along with 5 µL of o-dichlorobenzene-methanol solution (100 mg/L) as an internal standard. The headspace vial was equilibrated at 40 °C for 10 min, after which the extraction fiber was exposed to the headspace for 5 min. Finally, the SPME fiber was inserted into the GC inlet and desorbed at 240 °C for 5 min. The temperature program for GC was as follows: initial temperature of 38 °C held for 13 min, increased at a rate of 3 °C/min to 100 °C and held for 5 min, then increased at rates of 4 °C/min to 150 °C and 10 °C/min to 240 °C. The flow rate of carrier gas (He) was 1.0 mL/min. The MS conditions were as follows: Electron ionization (EI), transfer line temperature of 240 °C, ion source temperature of 240 °C, and mass scanning range of *m*/*z* 35–450. The detected volatile compounds were compared with the NIST17 spectral library for identification.

### 2.13 Electronic nose

According to the method of Zhang et al [25], an electronic nose system (PEN3, Airsense, Germany) equipped with an array of ten gas sensors was used to analyze the flavor changes in air-dried beef. The sensors included W1C (aromatic compounds), W5S (nitrogen oxides), W3C (ammonia, aromatic compounds), W6S (hydrides), W5C (short-chain alkanes), W1S (methyl groups, alkanes), W1W (inorganic sulfides), W2S (alcohols), W2W (organic sulfides), and W3S (long-chain alkanes). Two grams of ground sample were placed in a 20 mL headspace vial and heated in a 40 °C water bath for 8 min to facilitate subsequent analysis and achieve better response values. System parameters were as follows: cleaning time 120 s, sampling interval 1 s, data acquisition time 120 s, and chamber flow rate 400 mL/min.

### 2.14 Statistical analysis

One-way analysis of variance (ANOVA) was performed in SAS 8.0 (SAS Institute Inc., USA), and the means were compared by Duncan's multiple range test (*P*<0.05). Furthermore, heatmap was generated using R (Version 4.4.1, Tsinghua university, China) and RStudio (Version 2024.04.2 + 764, Posit Software, USA) to analyze the differences in volatile flavor compounds among various samples. The relative content of volatile flavor compounds was calculated by global standardization for taking the logarithm of the base 4 for all substances. All experiments were performed in triplicate.

## 3 Results and discussion

### 3.1 Color

Table 2 presents the color results of fresh (CT) and dried samples. Compared to the CT group (37.01), the L* values of the HAD group (35.57) exhibited a slight decrease (*P*<0.05), while significant increases (*P*<0.05) were noted in the other groups, ranging from 40.70 to 42.68. These changes may be attributed to alterations in the intrinsic fiber structure and moisture distribution of the beef. The increase in L* values is likely related to modifications in myofibrillar structure and the aggregation of sarcoplasmic proteins during thermal processing, which intensified light scattering in the beef [26]. The reduced lightness in the HAD group may result from a greater extent of the Maillard reaction, leading to melanin accumulation on the beef surface [25]. Furthermore, L* values also reflect moisture distribution; the penetration heating effect of IR and the homogenizing effect of ultrasound enhanced the uniformity of water distribution.

**Table 2:** Color and shear force of air-dried beef under different drying conditions.

Treatment	L*	a*	b*	Shear force (N)
CT	37.01 ± 0.99^c^	9.51 ± 0.72^a^	−2.88 ± 0.27^a^	/
HAD	35.57 ± 0.82^c^	3.44 ± 0.52^d^	−5.11 ± 0.34^c^	196.30 ± 10.12^a^
CU-HAD	41.77 ± 0.81^ab^	3.55 ± 0.35^cd^	−4.34 ± 0.41^b^	174.79 ± 7.19^b^
IRD	40.70 ± 0.91^b^	4.37 ± 0.44^bc^	−4.18 ± 0.46^b^	164.39 ± 9.30^b^
CU-IRD	42.68 ± 0.74^a^	5.05 ± 0.24^b^	−3.86 ± 0.52^b^	140.59 ± 13.95^c^

Note: Different letters in the same column indicate significant differences. CT stands for fresh samples. CT: fresh samples. HAD: hot air drying, CU-HAD: hot air drying with CU, IRD: hot air drying with IR, CU-IRD: hot air drying with CU and IR.

For a* and b* values, all samples exhibited significant declines (*P* <0.05). The CU-IRD group demonstrated the highest a* value of 5.05, while the HAD (3.44) and CU-HAD (3.55) groups showed the lowest values, possibly as a result of both drying time and the effects of ultrasonic cavitation. Lindahl et al [27] noted that the content of hemoglobin and the proportion of metmyoglobin were critical for the alteration of a* values. Continuous exposure to oxygen oxidized the iron ions in hemoglobin from the divalent to the trivalent state, leading to the conversion of myoglobin to metmyoglobin [28]. Additionally, free radicals formed by cavitation promoted lipid oxidation with increasing ultrasound time, generating large quantities of secondary products such as malondialdehyde [29]. Malondialdehyde can react with aldehyde groups on proximal and distal histidine residues of myoglobin, compromising its oxidative stability [30]. This mechanism may account for the relatively lower a* values observed in the CU-HAD group. Changes in b* values followed a similar trend to a* value, and previous research indicated that the form of myoglobin and internal reflectance were key factors influencing variations in b* values [31]. An increase in the MbO/Mb ratio and internal reflectance may visually manifest as an elevation in yellowness values.

### 3.2 Tenderness

The edible quality of meat products is directly correlated with their commercial value, with tenderness characteristics identified as critical factors influencing consumer choice and acceptance. Significant effects of CU and IR interventions during the drying process on shear force were observed (*P* <0.05), with a positive correlation established between shear force magnitude and drying time (Table 2). Local inhomogeneities in protein density and water retention within the beef muscle fiber network were found to increase as water migration rates decreased and drying times extended [32]. Proteins, fat, and connective tissues directly exposed to the drying medium were more prone to adhere to each other and form rigid structures compared to deeper tissue layers, which was known as the crust toughening or hardening [33]. The surface toughening effect, accompanying the coagulation and contraction of myofibrillar proteins, contributed to an increase in resistance to shear force [34]. In comparison to the HAD group, lower shear force values ranging from 140.59 N to 164.39 N were recorded for samples dried using IR, primarily attributed to reduced drying times. Furthermore, the high temperature and rapid drying characteristics of IR may induce protein hydrolysis reactions, weakening the compactness and stability of the muscle fiber structure.

Consistent with the findings of this study, positive effects of ultrasound on beef tenderization were reported by Kang et al [35], who attributed these effects to the cavitation and mechanical effects of ultrasound that disrupt the fibrous structure of muscle fibers. Concurrently, changes in the properties of connective tissue, including reduced mechanical strength and altered thermal stability of collagen, further compromised the structural integrity of myofibers. Additionally, Wang et al [36] revealed that ultrasonic treatment at 25 W/cm^2^ modulated the activity of calpain and promoted the degradation of desmin and troponin-T, leading to the structural disruption of muscle cells. Conversely, some studies reported no significant effects of ultrasonic tenderization, potentially due to short exposure times or insufficient ultrasonic intensity [37], [38].

### 3.3 Ultrastructure

**Fig. 2:**
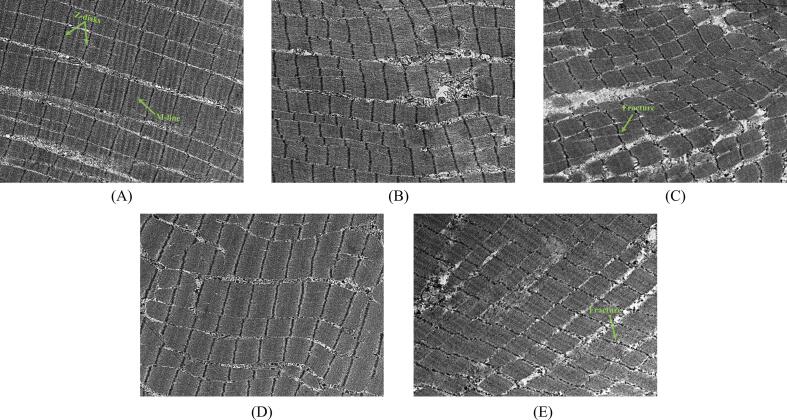
Ultrastructural images of samples under different conditions. Different letters (A-E) represent CT (fresh samples), HAD (hot air drying), CU-HAD (hot air drying with CU), IRD (hot air drying with IR) and CU-IRD (hot air drying with CU and IR) groups respectively.

The ultrastructure images of fresh and dried samples are illustrated in Fig. 2. The sarcomeres, as the fundamental unit of myofibrils, undergo alterations in its ultrastructure, specifically affecting the Z-disks and M-lines, following post-mortem denaturation and degradation of myofibrillar proteins [39]. In the CT group, sarcomeres were neatly aligned with clear distinctions between the A-bands and I-bands, and the Z-disks and M-lines remained intact. Heating caused the weakening or disappearance of the M-lines and A-bands in the samples, likely due to the thermal denaturation of myosin. Myosin, the primary component of thick filaments in the A-band, exists as double-stranded α-helices at low temperatures. The peptide chains within protein molecules are prone to rupture, leading to a transition from the original spatial conformation to β-sheets and random coils, thereby affecting the gel-forming ability of proteins [40].

In samples subjected to ultrasound treatment, myofibrils were visibly dispersed, with distortion and displacement of sarcomeres. Notably, transitional areas of sarcomeres, particularly around I-bands and Z-disks, exhibited fractures and filamentous structures, which was a sign of improved tenderness in some studies [36], [41]. One possible reason for this was the mechanical damage caused by the cavitation effect, affecting the extension of sarcomeres. Additionally, enzymatic effects were identified as other factors contributing to myofibrillar degradation. Got et al [42] applied ultrasound at 10 W/cm^2^ to bovine *semimembranosus* muscles and observed a 30 % increase of free Ca^2+^ content in the interstitial spaces, speculating that ultrasound accelerated the passive migration of Ca^2+^ from intracellular stores. High levels of Ca^2+^ entered the sarcoplasm and activated calcium-activated enzymes, which affected the hydrolysis rate of myofibrillar proteins and catalyzed the degradation of proteins at the Z-disks.

Sarcomere length is a critical metric for evaluating muscle contraction, influencing the material volume directly. The measured sarcomere lengths for each group were as follows: CT (1.610 ± 0.030 µm), HAD (1.344 ± 0.066 µm), CU-HAD (1.426 ± 0.076 µm), IRD (1.424 ± 0.031 µm), and CU-IRD (1.523 ± 0.027 µm), which aligned with the previous research [43]. The observed shortening of sarcomere length during the drying process might be attributed to irreversible changes in cytoskeletal proteins, particularly the coagulation of myosin and sarcoplasmic proteins alongside the dissipation of immobile water [44]. In contrast, the IRD and CU-IRD groups maintained more uniform sarcomere features due to reduced edge effects of temperature and moisture gradients imparted by IR. Among all treatment groups, the sarcomere length in the CU-IRD group was closest to the fresh samples and significantly higher than other treatment groups (*P* <0.05), indicating lower shrinkage rates in this group. Furthermore, a strong positive correlation between sarcomere length and tenderness has been established [45], suggesting its utility as a predictive tool for assessing changes in shear force.

### 3.4 Protein and fat oxidation

**Fig. 3:**
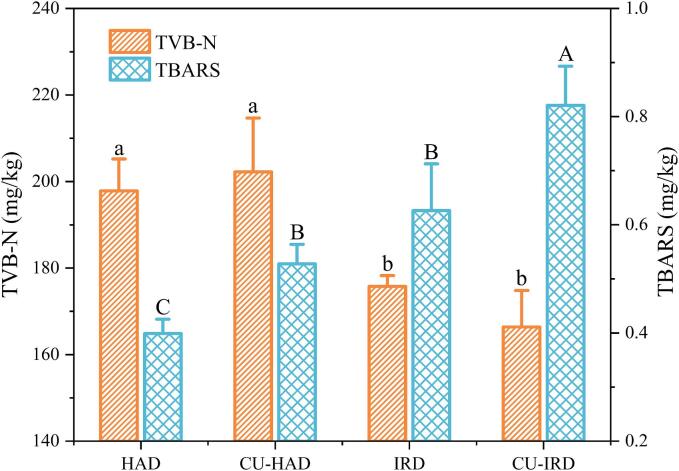
Oxidation of protein and fat in air-dried beef. Different letters, (a-b) and (A-C), indicate significant differences among different treatments for the TVB-N and TBARS values, respectively (*P*<0.05). HAD: hot air drying, CU-HAD: hot air drying with CU, IRD: hot air drying with IR, CU-IRD: hot air drying with CU and IR.

TVB-N is an alkaline nitrogenous compound, including ammonia and amines, produced in meat products through the breakdown of proteins by endogenous enzymes or extracellular enzymes from spoilage microorganisms. It has been utilized to assess the degree of oxidation and spoilage of proteins in certain fermented products [10]. The CU-IRD and IRD groups exhibited the lowest TVB-N values (Fig. 3) at 166.37 and 175.77 mg/kg, respectively, with no significant difference observed (*P*>0.05). The most severe protein oxidation was noted in the CU-HAD group, likely due to prolonged CU treatment, which generated a substantial number of aggressive free radicals, resulting in oxidative decarboxylation and deamidation of proteins [46].

Apart from the CU-HAD group, the variation in TVB-N was closely associated with drying time, decreasing as drying time was shortened. Prolonged drying was identified as a critical factor contributing to protein oxidation and spoilage, rather than elevated equilibrium temperature. Conversely, the mild conditions in the HAD and CU-HAD groups appeared to be more favorable. Krishnamurthy et al [47] suggested that IR could enhance energy absorption by certain spoilage microorganisms and associated enzymes, thereby increasing their lethality. Treatment of meatballs with infrared radiation at a heat flux of 3.7 to 8.5 kW/m^2^ resulted in a reduction of initial total mesophilic aerobic bacteria counts from 5.06 ± 0.02 log CFU/g to a range of 1.96–4.50 log CFU/g [48].

Fatty acids serve as primary flavor precursors generated by the hydrolysis of triglycerides and phospholipids. Most volatile compounds or flavor intermediates are derived from the oxidation of free fatty acids, which are subsequently utilized to form various other flavor substances through Maillard and other reactions [49]. In this process, certain polyunsaturated fatty acids (PUFAs) are particularly susceptible to attack by reactive oxygen species and radicals, decomposing into malondialdehyde (MDA), which is critical for reactions with TBA. Consequently, TBARS values (mg MDA/kg) are employed as an index of lipid oxidation [50]. The TBARS values of air-dried beef after various treatments ranged from 0.399 to 0.821 mg MDA/kg meat (Fig. 3), significantly below the 3.0 mg MDA/kg meat, which is the critical value for the development of off-flavors in fermented meat products [51].

Among all treatment groups, the IRD and CU-IRD groups exhibited significantly higher TBARS values compared to the HAD group (*P*<0.05), which may be related to the elevated internal equilibrium temperatures during the drying process. Different from HAD group, the increased thermal flux of IR reduced the temperature gradient within the material, resulting in enhanced fat hydrolysis and greater exposure of PUFAs. Consequently, PUFA-rich meats were theoretically more prone to oxidation, increasing aldehydes, ketones, or promoting Maillard reactions that influence flavor formation [52]. Additionally, ultrasound significantly enhanced fatty acid oxidation in the CU-HAD group, resulting in a 32.33 % increase in TBARS values compared to the HAD group. Zhang et al [53] reported a similar trend, attributing this effect to cavitation zones created by ultrasound, which operate under high temperature and pressure conditions, facilitating lipid hydrolysis and fatty acid oxidation. Notably, the presence of IR amplified the ultrasound effect, suggesting a synergistic interaction.

### 3.5 Protein aggregation and degradation

**Fig. 4:**
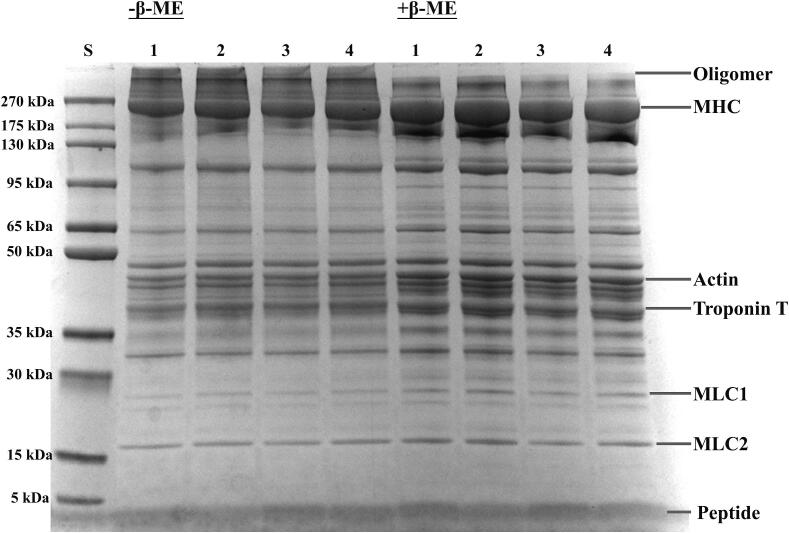
SDS-PAGE pattern of beef MP with different drying treatments. S: standard protein marker; The numbers (1–4) stand for HAD (hot air drying), CU-HAD (hot air drying with CU), IRD (hot air drying with IR) and CU-IRD (hot air drying with CU and IR) group, respectively.

Oxidation-induced changes in myofibrillar protein structure were observed through non-reduced (−β-ME) and reduced (+β-ME) gel electrophoresis. As illustrated in Fig. 4, all samples displayed characteristic myofibrillar protein (MP) profiles under both −β-ME and + β-ME conditions, including myosin heavy chain (MHC, 220 kDa), actin (42 kDa), troponin T (cTnT, 39 kDa), and myosin light chains (ML1, 22 kDa; ML2, 16 kDa) [54]. In the absence of β-ME, an unidentified high molecular weight soluble oligomer was detected at the top of the gel, attributed to the oxidation of sulfhydryl groups on two cysteine residues within the peptide chain, resulting in the formation of covalent bonds (S-S) and subsequent inter- or intramolecular covalent cross-linking and aggregation of protein molecules [20]. In the HAD and CU-HAD groups, the oligomers formed exhibited significantly darker bands, indicating a greater extent of oxidation.

Most oligomers were primarily composed of MHC, although other protein subunits were also present [55]. Unlike stable covalent bonds formed between amino acid residues, disulfide bonds function as dynamic chemical bonds that can be readily broken by reduction to sulfhydryl groups and subsequently re-oxidized. Treatment of myofibrillar proteins with the strong reducing agent β-ME resulted in the disruption of disulfide bonds and the depolymerization of oligomers, which facilitated the recovery of MHC bands and the deepening of bands corresponding to actin, cTnT, and ML1. Raghunath et al [56] found that high molecular weight protein components were more sensitive to heat, while drying had a minimal effect on low molecular weight protein constituents. Compared to the HAD and CU-HAD groups, air-dried beef after IR treatment showed narrower MHC bands, with reduced intensity in the bands for other components (actin, cTnT, ML1, and ML2). However, a thick band was observed below 5 kDa, which appeared significantly darker following IR treatment. This observation is hypothesized to result from infrared-accelerated thermal degradation of protein subunits, leading to the formation of lower molecular weight peptide fragments. Such thermal degradation is attributed to the attack of IR on covalent bonds, causing pronounced protein disintegration, which should be distinguished from the oxidation effects observed in the HAD and CU-HAD groups.

### 3.6 Free amino acids (FAAs)

Protein degradation is a representative feature of air-dried beef, and the formation of small peptides and FAAs play a crucial role in the taste of meat products. As depicted in Table 3, seventeen FAAs were identified in the samples subjected to various drying conditions. Compared to the HAD group (283.876 mg/100 g), composite drying treatments (CU-HAD, IRD, CU-IRD) increased the total amount of FAAs by 4.82 %, 8.57 %, and 18.03 %, respectively (*P*<0.05). Asp and Glu are major taste compounds contributing to umami flavor in air-dried beef, while Cys is associated with compounds that produce meaty flavor. The levels of Asp, Glu, and Cys in the CU-IRD group showed a significant increase (*P*<0.05), indicating enhanced flavor presentation.

**Table 3:** FAAs in air-dried beef.

Compounds (mg/100 g)	HAD	CU-HAD	IRD	CU-IRD
Asp	0.239 ± 0.020^a^	0.199 ± 0.019^b^	0.168 ± 0.006^b^	0.233 ± 0.019^a^
Glu	14.337 ± 0.328^c^	16.425 ± 0.172^b^	16.713 ± 0.078^b^	23.347 ± 0.662^a^
∑UAA	14.576 ± 0.310^c^	16.624 ± 0.190^b^	16.881 ± 0.072^b^	23.580 ± 0.681^a^
Thr	56.721 ± 3.298^b^	55.482 ± 1.214^b^	57.213 ± 2.175^b^	68.956 ± 3.090^a^
Ser	10.805 ± 0.564^b^	11.201 ± 0.139^ab^	11.795 ± 0.234^a^	11.668 ± 0.184^a^
Ala	55.631 ± 2.543^b^	57.211 ± 1.343^b^	58.396 ± 0.988^b^	65.269 ± 0.823^a^
Gly	25.827 ± 1.335^b^	25.455 ± 0.644^b^	26.538 ± 0.144^b^	28.944 ± 0.714^a^
Pro	10.442 ± 0.325^c^	12.016 ± 0.029^b^	12.172 ± 0.278^b^	15.868 ± 0.137^a^
∑SAA	159.426 ± 8.064^b^	161.366 ± 2.083^b^	166.113 ± 2.781^b^	190.704 ± 2.577^a^
Lys	11.009 ± 0.186^c^	12.197 ± 0.159^b^	13.472 ± 0.135^a^	12.156 ± 0.272^b^
Ile	10.326 ± 0.550^c^	11.136 ± 0.257^ab^	11.607 ± 0.137^a^	10.955 ± 0.086^b^
Val	16.606 ± 0.741^b^	17.806 ± 0.331^a^	18.355 ± 0.010^a^	18.073 ± 0.155^a^
Leu	18.744 ± 1.021^c^	20.585 ± 0.447^ab^	21.285 ± 0.268^a^	19.881 ± 0.129^b^
Tyr	10.851 ± 0.420^c^	11.888 ± 0.145^ab^	11.978 ± 0.088^a^	11.500 ± 0.090^b^
Phe	12.248 ± 0.715^b^	14.340 ± 0.563^a^	15.032 ± 0.303^a^	12.965 ± 0.145^b^
His	9.347 ± 0.473^c^	9.891 ± 0.308^bc^	10.486 ± 0.296^b^	11.644 ± 0.521^a^
Arg	11.033 ± 0.405^b^	11.469 ± 0.406^b^	12.616 ± 1.223^ab^	13.204 ± 0.916^a^
Met	5.945 ± 0.224^b^	6.632 ± 0.117^a^	6.830 ± 0.100^a^	5.706 ± 0.042^b^
∑BAA	106.110 ± 4.206^c^	115.943 ± 2.400^b^	121.660 ± 2.527^a^	116.083 ± 1.660^b^
Cys	3.764 ± 0.085^b^	3.640 ± 0.166^b^	4.050 ± 0.323^b^	4.685 ± 0.266^a^
∑OAA	3.764 ± 0.085^b^	3.640 ± 0.166^b^	4.050 ± 0.323^b^	4.685 ± 0.266^a^
∑FAA	283.876 ± 12.491^c^	297.573 ± 4.346^b^	308.704 ± 0.494^b^	335.052 ± 3.038^a^
∑UAA/∑FAA	0.051 ± 0.001^c^	0.056 ± 0.001^b^	0.055 ± 0.000^b^	0.070 ± 0.002^a^
∑SAA/∑FAA	0.561 ± 0.004^a^	0.542 ± 0.002^b^	0.538 ± 0.008^b^	0.569 ± 0.003^a^
∑BAA/∑FAA	0.374 ± 0.002^b^	0.390 ± 0.003^a^	0.394 ± 0.009^a^	0.346 ± 0.006^c^
∑OAA /∑FAA	0.013 ± 0.001^ab^	0.012 ± 0.001^b^	0.013 ± 0.001^ab^	0.014 ± 0.001^a^

Note: The superscripts with different letters (a, b and c) in the same row indicated the significant difference at *P*<0.05. HAD: hot air drying, CU-HAD: hot air drying with CU, IRD: hot air drying with IR, CU-IRD: hot air drying with CU and IR. UAA: umami amino acids (Asp, Glu); SAA: sweet amino acids (Thr, Ser, Ala, Gly, Pro); BAA: bitter amino acids (Lys, Ile, Val, Leu, Tyr, Phe, His, Arg, Met); OAA: other amino acids (Cys).

No significant differences (*P*>0.05) in other FAAs were observed between the CU-HAD and IRD groups, except for Lys. Previous research indicated significant differences in heat transfer between the CU-HAD and IRD groups (*P*<0.05), resulting in stable internal drying temperatures of 33 °C and 38 °C, respectively [14]. Therefore, we hypothesized that the mechanisms of protein degradation by CU and IR came from two distinct levels. Shi et al [57] reported that different external environments (light, oxygen, temperature, etc.) led to different protein hydrolysis and release of flavor-related FAAs. In the IRD group, the heating properties of infrared (IR) and the spectral absorption of protein groups facilitated thermal degradation. The peptide bonds (N–C=O) and other covalent bonds (–OH, N–H) that maintained the primary structure of proteins absorbed infrared energy and gradually broke, causing myofibrillar proteins to lose structural stability and begin to degrade [58]. The degradation of proteins by CU, on the other hand, may originate from prolonged cutting and oxidation of proteins [59]. The high-speed microjets, agitation, and strongly oxidative hydroxyl radicals produced by the collapse of cavitation bubbles result in structural changes and oxidative degradation of proteins [60].

Notably, the content of bitter amino acids (BAA) in the IRD group was significantly higher than in other groups (*P* <0.05), while the contents of umami amino acids (UAA) and sweet amino acids (SAA) decreased, indicating that prolonged IR treatments enhanced the bitterness of air-dried beef. Liu et al [61] explained this phenomenon by noting that the Maillard reaction accelerates the consumption of some FAAs, leading to the formation and release of aldehydes, sulfur-containing compounds, and nitrogen-containing compounds. During this process, some non-polar amino acids (Val, Leu, Ile, Met) required higher energy for Maillard reactions and were thus less likely to be consumed [62]. Both CU and IR treatments significantly increased the proportion of UAA. However, individual applications of CU or IR may result in decreased SAA proportions and increased BAA proportions, which is undesirable. In contrast, the CU-IRD group exhibited a favorable distribution of flavor amino acids, characterized by the highest ratios of UAA and SAA and the lowest ratio of BAA. Therefore, this combined treatment is considered beneficial for regulating the distribution of flavor amino acids.

### 3.7 Fatty acid composition

Free fatty acids (FFAs) primarily arise from the hydrolysis and thermal degradation of triglycerides and phospholipids during processing, representing initial steps in the conversion of lipids into flavor compounds in beef [63]. As specialized flavor precursors, FFAs further engage in oxidation processes (both autoxidation and enzymatic oxidation) during thermal treatment, leading to the formation of hydroperoxides. These hydroperoxides are secondarily oxidized through a series of complex reactions and decomposed into small molecule volatile compounds (e.g. aldehydes, alcohols) [64]. The types and concentrations of FFAs in air-dried beef are summarized in Table 4. A total of 17 FFAs were identified, comprising six saturated fatty acids (SFAs), seven monounsaturated fatty acids (MUFAs), and four polyunsaturated fatty acids (PUFAs). The samples were predominantly composed of SFAs (approximately 50.674 %), with palmitic acid (C16:0) and stearic acid (C18:0) identified as the major fatty acids, consistent with findings reported by Kim et al [65].

**Table 4:** Effect of different drying treatments on fatty acid composition and content of air-dried beef.

Fatty acid types(mg/100 g)	HAD	CU-HAD	IRD	CU-IRD
C13:0	3.226 ± 0.700^d^	6.385 ± 0.083^c^	12.230 ± 1.444^b^	25.527 ± 0.710^a^
C14:0	11.001 ± 2.169^b^	15.303 ± 3.946^b^	16.259 ± 1.094^b^	27.549 ± 3.604^a^
C14:1	7.738 ± 3.144^c^	20.850 ± 3.724^c^	35.773 ± 1.779^b^	62.291 ± 13.994^a^
C15:0	4.242 ± 1.684^a^	5.685 ± 3.748^a^	5.199 ± 0.595^a^	3.090 ± 3.150^a^
C15:1	3.162 ± 0.427^d^	5.862 ± 0.539^c^	10.180 ± 0.388^b^	16.295 ± 1.866^a^
C16:0	320.197 ± 2.154^c^	391.658 ± 0.554^b^	395.233 ± 1.451^b^	586.962 ± 55.553^a^
C16:1	21.836 ± 0.139^a^	26.408 ± 0.256^a^	23.501 ± 0.668^a^	24.909 ± 6.543^a^
C17:1	6.107 ± 0.337^d^	11.019 ± 0.527^c^	15.987 ± 2.386^b^	27.748 ± 3.368^a^
C18:0	399.358 ± 0.553^c^	499.497 ± 2.221^b^	530.102 ± 6.766^b^	687.010 ± 45.358^a^
C18:1n9t	38.757 ± 29.462^b^	70.985 ± 4.176^a^	77.042 ± 2.494^a^	88.912 ± 5.546^a^
C18:1n9c	454.489 ± 1.254^c^	543.808 ± 4.821^ab^	515.200 ± 4.183^b^	582.015 ± 58.330^a^
C18:2n6t	1.924 ± 0.096^b^	2.289 ± 0.319^b^	1.409 ± 0.630^b^	3.658 ± 1.002^a^
C18:2n6c	233.045 ± 1.013^bc^	260.453 ± 2.885^a^	249.017 ± 3.705^ab^	224.997 ± 18.591^c^
C20:1	8.719 ± 0.928^a^	9.373 ± 0.216^a^	8.711 ± 0.949^a^	9.896 ± 2.227^a^
C20:3n6	11.558 ± 0.284^a^	11.046 ± 0.209^a^	10.219 ± 1.971^a^	0
C20:4n6	61.406 ± 1.174^a^	59.674 ± 1.212^a^	61.311 ± 2.582^a^	54.074 ± 4.311^b^
C23:0	3.334 ± 0.427^c^	4.366 ± 1.731^c^	9.892 ± 1.461^b^	36.586 ± 5.122^a^
∑SFA	741.359 ± 4.142^c^	922.893 ± 10.281^b^	968.916 ± 11.600^b^	1407.333 ± 130.188^a^
∑MUFA	540.808 ± 25.065^c^	688.305 ± 5.826^b^	686.394 ± 9.038^b^	812.064 ± 68.190^a^
∑PUFA	307.933 ± 2.532^b^	333.461 ± 4.353^a^	321.956 ± 5.758^ab^	282.729 ± 22.557^c^
∑FFA	1590.100 ± 19.038^c^	1944.659 ± 19.621^b^	1977.265 ± 26.190^b^	2461.517 ± 186.197^a^
∑PUFA /∑SFA	0.415 ± 0.002^a^	0.361 ± 0.002^b^	0.332 ± 0.002^c^	0.201 ± 0.004^d^

Note: The superscripts with different letters (a, b and c) in the same row indicated the significant difference at *P* <0.05. HAD: hot air drying, CU-HAD: hot air drying with CU, IRD: hot air drying with IR, CU-IRD: hot air drying with CU and IR. SFA: saturated fatty acids, MUFA: monounsaturated fatty acids, PUFA: polyunsaturated fatty acids, FFA: free fatty acids.

Medium and long-chain unsaturated FFAs can serve as substrates for further oxidative degradation, generating small volatile compounds. The variation in FFAs is influenced by the balance between hydrolytic production and oxidative consumption. Evidently, the CU-IRD group demonstrated the highest accumulation of FFAs (2461.517 mg/100 g), with significantly elevated levels of SFAs (1407.333 mg/100 g) and MUFAs (812.064 mg/100 g) compared to other groups (*P* <0.05). The presence of MUFAs is associated with improvements in flavor, tenderness, and juiciness of meat [66], so it can be assumed that the samples from the CU-IRD group had a higher overall acceptability for consumers. Conversely, the CU-IRD group displayed the lowest level of PUFAs (282.729 mg/100 g) (*P* <0.05). On the contrary, the PUFAs in the CU-HAD and IRD group increased by 8.29 % (*P*<0.05) and 4.55 % (*P* >0.05), respectively, compared to HAD.

The differences in lipid hydrolysis and oxidation are proposed as significant factors contributing to variations in FFA profiles. It is evident that both CU and IR treatments substantially promoted the accumulation of FFAs. Rejasse et al [67] revealed that lipases and phospholipases related to lipolysis (such as acidic lipase, neutral lipase, and phospholipase) enhanced catalytic efficiency (temperature and enzyme activity) under the effect of microwave and infrared radiations, facilitating the hydrolysis of lipids. Additional studies have highlighted the beneficial effects of ultrasound on lipase catalysis [68], [69], attributing these effects to the cavitation phenomenon, which alters temperature and pressure in the microenvironment, enhances substrate solubility, modifies protein conformation, and boosts enzyme activity. Furthermore, the mechanical disruption of muscle cells by ultrasound promoted the release of hydrolytic enzymes and cofactors (Ca^2+^), thereby increasing the contact area between enzymes and substrates.

Compared to SFAs and MUFAs, the double bonds in PUFAs weaken the C-H bonds connected to them, making the hydrogen atoms on the methylene group relatively reactive. Due to the larger size of the molecules, these groups are more susceptible to attacked by oxygen radicals [70]. The observed reduction in PUFAs within the CU-IRD group was attributed to oxidative consumption surpassing hydrolytic production, particularly for C20:3n6 and C20:4n6, and the combined treatment with CU and IR promoted a heightened tendency for oxidation. Theoretically, an increased degree of oxidative degradation of unsaturated fatty acids facilitates the release of key volatile compounds that enhance meat aroma, including aldehydes, alcohols, and ketones [71]. However, the resultant decrease in the ratio of ∑PUFA/∑SFA may not align with recommendations from international dietary authorities [72]. In contrast, the CU-HAD group exhibited the least reduction in the ∑PUFA/∑SFA ratio, likely due to the comparatively lower consumption of PUFAs by CU relative to IR.

### 3.8 Electronic tongue

**Fig. 5:**
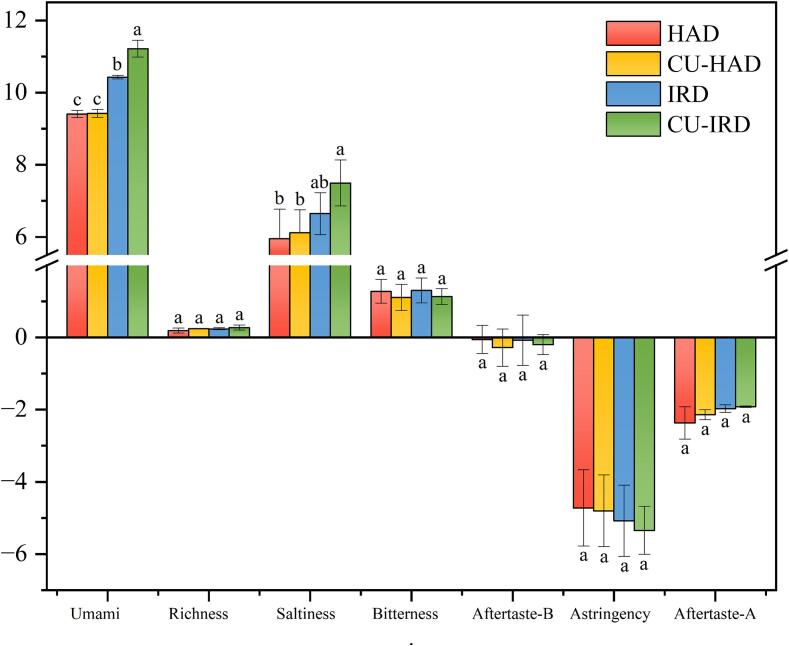
Evolution on taste of electronic tongue during different drying processes. Different letters (a-c) for the same parameter indicate significant differences (*P* <0.05) between treatments. HAD: hot air drying, CU-HAD: hot air drying with CU, IRD: hot air drying with IR, CU-IRD: hot air drying with CU and IR.

The electronic tongue is utilized to convert electrical signals into taste signals, effectively characterizing the non-volatile taste components in foods and accurately reflecting the diverse tastes of various samples. Its low sensory threshold allows for the elimination of subjectivity in sensory evaluations [10]. The output results for taste characteristics are presented in Fig. 5. Since the taste characteristic values for sourness were significantly less than zero (−29.997 to −24.313), it was determined that this taste did not influence the overall sensory profile. Therefore, it was excluded from Fig. 5. Results obtained from the electronic tongue indicated significant differences in umami and saltiness among groups (*P* <0.05), while trends for bitterness and astringency were observed but not statistically significant (*P* >0.05). Among the taste receptors on the electronic tongue, umami consistently ranked highest, primarily attributed to the contribution of Glu. Although saltiness is induced by Na^+^, Tian et al [73] noted that some umami amino acids (such as Asp and Glu), play a critical role in the perception of saltiness. These umami amino acids may serve as alternatives to reduce sodium content while maintaining saltiness. Consistent with findings related to FAAs, the CU-IRD group exhibited the highest levels of umami (11.217) and saltiness (7.497), indicating more desirable taste characteristics. Bitterness is a common sensory defect in air-dried beef. In the electronic tongue scoring, the values of the bitter response and the trend of the different treatment groups differed from their bitter amino acid content, which could be interfered by other presenting flavors (sweet amino acids).

### 3.9 Volatile flavor compounds

**Fig. 6:**
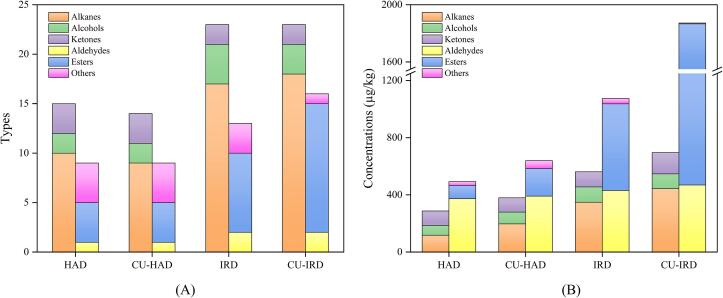
Profiles of volatile flavor compounds for different drying methods. (A), (B) represent the effect of drying methods on the types and concentrations of different volatile flavor substances, respectively. HAD: hot air drying, CU-HAD: hot air drying with CU, IRD: hot air drying with IR, CU-IRD: hot air drying with CU and IR.

The development of flavor in meat products requires a series of interconnected chemical changes, including air oxidation, Maillard reactions, Strecker degradation, and enzymatic degradation [74]. Important flavor compounds function as precursors or intermediates, engaging in interactions with existing substances or contributing to the formation of specific flavor compounds. Therefore, the aroma of food is not solely the result of individual compounds. Comprehensive scanning analysis of samples using HPSE-GC/MS technology revealed the detection of 64 volatile compounds, comprising 27 alkanes, 5 alcohols, 4 ketones, 3 aldehydes, 18 esters, and 7 other compounds. The types and concentrations of volatile compounds are depicted in Fig. 6. The volatile flavor components of air-dried beef were predominantly composed of esters, aldehydes, and alkanes, which accounted for 38.10 %, 27.75 %, and 18.46 % of the total substance content, respectively. The CU-IRD group exhibited increased diversity (39 substances) and total content (2568.10 µg/kg) of volatile flavor compounds compared to other groups. The incorporation of IR and CU treatments positively influenced flavor, primarily through alterations in the variety and concentration of certain alkanes and esters.

**Fig. 7:**
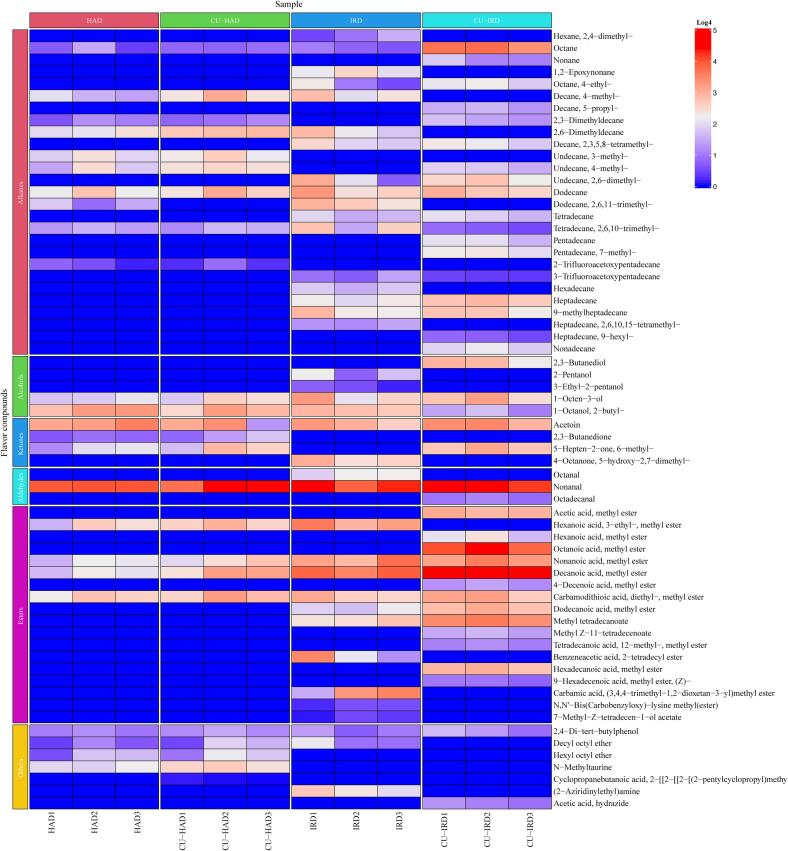
Visual heatmap of volatile flavor compounds in air-dried meat. HAD: hot air drying, CU-HAD: hot air drying with CU, IRD: hot air drying with IR, CU-IRD: hot air drying with CU and IR.

As illustrated in Fig. 7, CU and IR increased the formation of alkanes in the air-dried beef, including straight-chain and branched-chain alkanes. Aliphatic and aromatic alkanes are products of lipid oxidation, and their levels correlate with the degree of lipid oxidation in beef. Straight-chain alkanes may exert less impact on flavor due to their relatively high flavor thresholds, such as octane and dodecane [75]. However, some branched-chain alkanes still possess certain flavors and may contribute to the overall flavor profiles.

Alcohols are typically derived from the reduction of methyl ketones, lipid oxidation, and amino acid decomposition. In all groups, 1-octen-3-ol and 2-butyl-1-octanol were detected. The 1-octen-3-ol (mushroom flavor) produced by oxidation of arachidonic acid is an unsaturated alcohol with a low flavor threshold (1 µg/kg) and can be considered as an important contributor to air-dried beef flavour [76]. Additionally, a greater diversity of alcohol compounds, including 3-ethyl-2-pentanol, 2-pentanol, and 2,3-butanediol, was observed in the IRD and CU-IRD groups. The 2,3-butanediol is recognized as a major alcohols produced in dry-cured meat (fermented meat products) [77], possibly due to enhanced Strecker degradation induced by IR. Compared to the IRD group, the CU-IRD group presented a reduction in both the variety and quantity of alcohols, attributed to their depletion through esterification reactions with carboxylic acids. However, saturated alcohols generally possess higher flavor thresholds, suggesting that these lost alcohols may have a minor impact on flavor.

Ketones mainly arise from lipid oxidation or amino acid degradation in meat products and serve as important intermediates for heterocyclic aromatic compounds [78], such as acetoin (with a distinctive buttery aroma), 2,3-butanedione (with a strong creamy, fermented, creamy, and sweet flavor), and 6-methyl-5-hepten-2-one (resembling apple, banana, and butter flavors). The acetoin (from 60.86 to 96.85 µg/kg) is the predominant ketone formed during drying, with a low flavor threshold, indicating its significant contribution to meat flavor. Throughout the drying process, the CU-IRD group accumulated the highest levels of ketones, consistent with the TBARS results.

Aldehydes are primarily generated during the oxidation of PUFAs and Strecker degradation of some amino acids, typically possessing lower flavor thresholds than alcohols [79]. Nonanal was the most abundant volatile flavor compound with concentrations of 373.74, 391.49, 430.94, and 469.93 g/kg across all groups, indicating active fat oxidation during air drying of beef. CU and IR treatments undoubtedly enhanced the β-oxidation of PUFAs, potentially due to the action of microorganisms and enzymes. Previous study has also identified nonanal in dry-cured meat, highlighting its contribution to the sensory quality through its strong greasy odor and rapid release during the drying process, which may have additive effects on other flavor compounds [80].

Esters are produced through esterification reactions between carboxylic acids and alcohols, with multiple formation pathways including protein hydrolysis, fat oxidation, glycolysis, and the action of endogenous enzymes [81]. A total of 18 esters were detected across the four groups. Compounds such as methyl acetate (fragrant fruity flavor), methyl hexanoate (fruity, bacon-like aroma), methyl octanoate (strong fruity, orange-like aroma), methyl nonanoate (pear, wine-like flavor), methyl decanoate (wine-like, fruity, floral odors), and methyl dodecanoate (coconut, mushroom-like flavors) exhibit strong flavor characteristics that positively influence air-dried beef. The CU-IRD and IRD groups demonstrated higher contents of esters than other groups, particularly methyl octanoate, methyl decanoate, and methyl nonanoate. It has been noted that microwaves enhanced the efficiency of esterification reactions in biodiesel production, primarily due to reduced heating times and activation energy from thermal effects [82]. According to our speculation, the effects similar to microwave can be achieved with IR.

### 3.10 Electronic nose

**Fig. 8:**
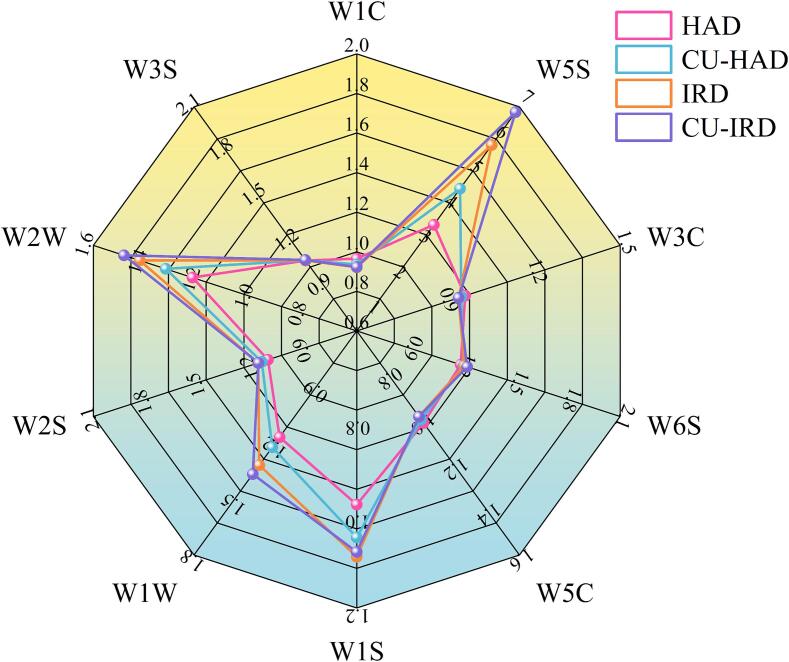
Radar plot of the response by electronic nose for different samples. HAD: hot air drying, CU-HAD: hot air drying with CU, IRD: hot air drying with IR, CU-IRD: hot air drying with CU and IR.

The electronic nose system is capable of mimicking the human olfactory system, allowing for the distinction of subtle changes in odors. The radargram of the electronic nose for air-dried beef is presented in Fig. 8. Sensors W5S, W1S, W1W, and W2W exhibited strong response values, indicating that nitrogen oxides, methyl compounds (alkanes), and sulfides were the primary odor compounds. Zhong et al [25] noted that nitrogen oxides and sulfides serve as representative compounds for the meaty and roasted aromas of meat products, with higher values correlating with a stronger meaty and roasted aroma. It can be concluded that CU and IR treatments effectively preserved traditional air-dried beef flavors while enhancing the meaty aroma. In the CU-IRD group, the highest response values were recorded for sensors W5S, W1W, and W2W, possibly due to synergistic effects from these two techniques, which intensified the thermal degradation of proteins and amino acids. Additionally, W1S can detect lipid degradation products (primarily methyl alkanes), and the results indicated that beef in IRD and CU-IRD group underwent relatively higher lipid oxidation during processing, consistent with TBARS results.

## 4 Conclusions

The present study demonstrated that contact ultrasound (CU) and infrared radiation (IR) assisted hot air drying is an effective processing method for air-dried beef, mitigating issues such as crust hardening, excessive browning, and flavor loss during processing. A significant reduction in drying time was achieved with IR, leading to improved color in the samples. Notable effects on tenderness and ultrastructure were observed with CU, primarily attributed to mechanical damage from cavitation effects, which resulted in Z-disk disruption. Compared to the hot air drying (HAD) group, CU and IR facilitated the hydrolysis of fats and proteins, evidenced by the release of free amino acids (FAAs) and free fatty acids (FFAs). Analyses using TBARS, GC–MS, and electronic nose techniques indicated that these methods enhanced the production of volatile compounds from lipid oxidation, imparting a richer aroma to the air-dried beef (roasted, fruity, and greasy odour). In conclusion, this paper demonstrated that CU-IRD represents a potentially effective technology for improving the quality of air-dried beef, offering theoretical insights into the mechanisms of quality enhancement and flavor development.

## CRediT authorship contribution statement

**Jiahua Gao:** Writing – review & editing, Writing – original draft, Visualization, Validation, Supervision, Software, Resources, Project administration, Methodology, Investigation, Funding acquisition, Formal analysis, Data curation, Conceptualization. **Siyu Cheng:** Data curation, Conceptualization. **Xiaomei Sun:** Software. **Yun Bai:** Funding acquisition. **Xiaobo Yu:** Funding acquisition. **Xianming Zeng:** Supervision, Software, Resources. **Songmei Hu:** Funding acquisition. **Minwei Zhang:** Formal analysis, Data curation. **Jianping Yue:** Data curation. **Xinglian Xu:** Software, Resources, Project administration. **Minyi Han:** Writing – review & editing, Project administration.

## Declaration of competing interest

The authors declare that they have no known competing financial interests or personal relationships that could have appeared to influence the work reported in this paper.

## References

[1] Kim S.-M., Kim T.-K., Cha J.Y. (2021). Novel processing technologies for improving quality and storage stability of jerky: A review. LWT Food Sci. Technol..

[2] Rao W., Wang Z., Li G. (2020). Formation of crust of dried meat and its relationship to moisture migration during air drying. J. Food Process. Preserv..

[3] Kaveh M., Abbaspour-Gilandeh Y., Taghinezhad E. (2021). The quality of infrared rotary dried terebinth (*pistacia atlantica l.*)-optimization and prediction approach using response surface methodology. Molecules.

[4] Obajemihi O.I., Cheng J.-H., Sun D.-W. (2023). Novel sequential and simultaneous infrared-accelerated drying technologies for the food industry: Principles, applications and challenges. Crit. Rev. Food Sci. Nutr..

[5] Zheng Z., Zhang M., Fan H. (2021). Effect of microwave combined with ultrasonic pretreatment on flavor and antioxidant activity of hydrolysates based on enzymatic hydrolysis of bovine bone. Food Biosci..

[6] Liu S., Yin H., Pickard M. (2020). Influence of infrared heating on the functional properties of processed lentil flours: A study focusing on tempering period and seed size. Food Res. Int..

[7] Yu J., Wang M., Zhang M. (2021). Effect of infrared ray roasting on oxidation stability and flavor of virgin rapeseed oils. J. Food Sci..

[8] Bozkir H., Tekgül Y., Erten E.S. (2021). Effects of tray drying, vacuum infrared drying, and vacuum microwave drying techniques on quality characteristics and aroma profile of orange peels. J. Food Process Eng.

[9] Xu L., Zheng Y., Zhou C. (2021). Kinetic response of conformational variation of duck liver globular protein to ultrasonic stimulation and its impact on the binding behavior of *n*-alkenals. LWT Food Sci. Technol..

[10] Bao G., Niu J., Li S. (2022). Effects of ultrasound pretreatment on the quality, nutrients and volatile compounds of dry-cured yak meat. Ultrason. Sonochem..

[11] Chang H.-J., Wang Q., Tang C.-H. (2015). Effects of ultrasound treatment on connective tissue collagen and meat quality of beef *semitendinosus* muscle. J. Food Qual..

[12] Hu Y., Dong Z., Wen R. (2023). Combination of ultrasound treatment and starter culture for improving the quality of beef jerky. Meat Sci..

[13] Zou Y., Kang D., Liu R. (2018). Effects of ultrasonic assisted cooking on the chemical profiles of taste and flavor of spiced beef. Ultrason. Sonochem..

[14] Gao J., Cheng S., Zeng X. (2024). Effects of contact ultrasound coupled with infrared radiation on drying kinetics, water migration and physical properties of beef during hot air drying. Ultrason. Sonochem..

[15] Sánchez-Torres E.A., Abril B., Benedito J. (2022). Airborne ultrasonic application on hot air-drying of pork liver. Intensification of moisture transport and impact on protein solubility. Ultrason. Sonochem..

[16] Mason T.J., Lorimer J.P., Bates D.M. (1992). Quantifying sonochemistry: Casting some light on a ‘black art’. Ultrasonics.

[17] Luo J., Xu W., Liu Q. (2022). Dielectric barrier discharge cold plasma treatment of pork loin: Effects on muscle physicochemical properties and emulsifying properties of pork myofibrillar protein. LWT Food Sci. Technol..

[18] Gharibzahedi S.M.T., Mohammadnabi S. (2017). Effect of novel bioactive edible coatings based on jujube gum and nettle oil-loaded nanoemulsions on the shelf-life of Beluga sturgeon fillets. Int. J. Biol. Macromol..

[19] Luo X., Xiao S., Ruan Q. (2022). Differences in flavor characteristics of frozen surimi products reheated by microwave, water boiling, steaming, and frying. Food Chem..

[20] Kang D., Zou Y., Cheng Y. (2016). Effects of power ultrasound on oxidation and structure of beef proteins during curing processing. Ultrason. Sonochem..

[21] Gornall A.G., Bardawill C.J., David M.M. (1949). Determination of serum proteins by means of the biuret reaction. J. Biol. Chem..

[22] Barido F.H., Kim H.-J., Shin D.-J. (2022). Physicochemical characteristics and flavor-related compounds of fresh and frozen-thawed thigh meats from chickens. Foods.

[23] Wang D., Zhang J., Zhu Z. (2022). Effect of ageing time on the flavour compounds in Nanjing water-boiled salted duck detected by HS-GC-IMS. LWT Food Sci. Technol..

[24] Xu Y., Zhang D., Chen R. (2021). Comprehensive evaluation of flavor in charcoal and electric-roasted *tamarix* lamb by HS-SPME/GC-MS combined with electronic tongue and electronic nose. Foods.

[25] Zhang W.-K., Zhang C., Qi B. (2023). Hot-air impingement roast drying of beef jerky: Effect of relative humidity on quality attributes. Drying Technol..

[26] Purslow P.P., Warner R.D., Clarke F.M. (2020). Variations in meat colour due to factors other than myoglobin chemistry; a synthesis of recent findings (invited review). Meat Sci..

[27] Lindahl G., Lundström K., Tornberg E. (2001). Contribution of pigment content, myoglobin forms and internal reflectance to the colour of pork loin and ham from pure breed pigs. Meat Sci..

[28] Mewa E.A., Okoth M.W., Kunyanga C.N. (2018). Effect of drying air temperature and slice thickness on the physical and microbiological quality of dried beef. LWT Food Sci. Technol..

[29] Cao C., Xiao Z., Tong H. (2021). Effect of ultrasound-assisted enzyme treatment on the quality of chicken breast meat. Food Bioprod. Process..

[30] Ramanathan R., Suman S.P., Faustman C. (2020). Biomolecular interactions governing fresh meat color in post-mortem skeletal muscle: A review. J. Agric. Food Chem..

[31] Tam L.G., Berg E.P., Gerrard D.E. (1998). Effect of halothane genotype on porcine meat quality and myoglobin autoxidation. Meat Sci..

[32] Palka K., Daun H. (1999). Changes in texture, cooking losses, and myofibrillar structure of bovine *M. semitendinosus* during heating. Meat Sci..

[33] Bai Y., Rahman M.S., Perera C.O. (2002). Structural changes in apple rings during convection air-drying with controlled temperature and humidity. J. Agric. Food Chem..

[34] Becker A., Boulaaba A., Pingen S. (2016). Low temperature cooking of pork meat — physicochemical and sensory aspects. Meat Sci..

[35] Kang D., Gao X., Ge Q. (2017). Effects of ultrasound on the beef structure and water distribution during curing through protein degradation and modification. Ultrason. Sonochem..

[36] Wang A., Kang D., Zhang W. (2018). Changes in calpain activity, protein degradation and microstructure of beef *M. semitendinosus* by the application of ultrasound. Food Chem..

[37] Lyng J.G., Allen P., McKenna B.M. (1998). The effect on aspects of beef tenderness of pre- and post-rigor exposure to a high intensity ultrasound probe. J. Sci. Food Agric..

[38] Pohlman F.W., Dikeman M.E., Zayas J.F. (1997). The effect of low-intensity ultrasound treatment on shear properties, color stability and shelf-life of vacuum-packaged beef semitendinosus and biceps femoris muscles. Meat Sci..

[39] Koohmaraie M., Geesink G.H. (2006). Contribution of postmortem muscle biochemistry to the delivery of consistent meat quality with particular focus on the calpain system. Meat Sci..

[40] Vaskoska R., Ha M., Ong L. (2021). Myosin sensitivity to thermal denaturation explains differences in water loss and shrinkage during cooking in muscles of distinct fibre types. Meat Sci..

[41] Lee E.Y., Rathnayake D., Son Y.M. (2023). Effect of novel high-intensity ultrasound technique on physio-chemical, sensory attributes, and microstructure of bovine *semitendinosus* muscle. Food Science of Animal Resources.

[42] Got F., Culioli J., Berge P. (1999). Effects of high-intensity high-frequency ultrasound on ageing rate, ultrastructure and some physico-chemical properties of beef. Meat Sci..

[43] Astruc T., Gatellier P., Labas R. (2010). Microstructural changes in *m. rectus abdominis* bovine muscle after heating. Meat Sci..

[44] Palka K. (2003). The influence of post-mortem ageing and roasting on the microstructure, texture and collagen solubility of bovine *semitendinosus* muscle. Meat Sci..

[45] Battaglia C., Vilella G.F., Bernardo A.P.S. (2020). Comparison of methods for measuring shear force and sarcomere length and their relationship with sensorial tenderness of longissimus muscle in beef. J. Texture Stud..

[46] Zhang J., Zhang Y., Zou Y. (2021). Effects of ultrasound-assisted cooking on quality characteristics of spiced beef during cold storage. LWT Food Sci. Technol..

[47] Krishnamurthy K., Khurana H.K., Soojin J. (2008). Infrared heating in food processing: An overview. Compr. Rev. Food Sci. Food Saf..

[48] Sengun I.Y., Icier F., Kor G. (2017). Effects of combined ohmic–infrared cooking treatment on microbiological inactivation of meatballs. J. Food Process Eng.

[49] Bai S., Wang Y., Luo R. (2021). Formation of flavor volatile compounds at different processing stages of household stir-frying mutton sao zi in the northwest of China. LWT Food Sci. Technol..

[50] Ojha K.S., Harrison S.M., Brunton N.P. (2017). Statistical approaches to access the effect of *Lactobacillus sakei* culture and ultrasound frequency on fatty acid profile of beef jerky. J. Food Compos. Anal..

[51] Wójciak K.M., Stasiak D.M., Ferysiuk K. (2019). The influence of sonication on the oxidative stability and nutritional value of organic dry-fermented beef. Meat Sci..

[52] Ames J.M., Guy R.C.E., Kipping G.J. (2001). Effect of pH, temperature, and moisture on the formation of volatile compounds in glycine/glucose model systems. J. Agric. Food Chem..

[53] Zhang J., Zhang Y., Wang Y. (2020). Influences of ultrasonic-assisted frying on the flavor characteristics of fried meatballs. Innov. Food Sci. Emerg. Technol..

[54] Sun Q., Chen Q., Xia X. (2019). Effects of ultrasound-assisted freezing at different power levels on the structure and thermal stability of common carp (*Cyprinus carpio*) proteins. Ultrason. Sonochem..

[55] Liu H., Zhang H., Liu Q. (2020). Solubilization and stable dispersion of myofibrillar proteins in water through the destruction and inhibition of the assembly of filaments using high-intensity ultrasound. Ultrason. Sonochem..

[56] Raghunath M.R., Sankar T.V., Ammu K. (1995). Biochemical and nutritional changes in fish proteins during drying. J. Sci. Food Agric..

[57] Shi S., Kong B., Wang Y. (2020). Comparison of the quality of beef jerky processed by traditional and modern drying methods from different districts in Inner Mongolia. Meat Sci..

[58] Chen Q., Zhang Y., Jing L. (2022). Changes in protein degradation and non-volatile flavor substances of swimming crab (*portunus trituberculatus*) during steaming. Foods.

[59] Zhou C.-Y., Xia Q., He J. (2022). Insights into ultrasonic treatment on the mechanism of proteolysis and taste improvement of defective dry-cured ham. Food Chem..

[60] Lin W., Zhu J., Sun Y. (2024). Effects of ultrasonic-assisted marinating on degradation of beef protein and formation of flavor precursors. J. Food Compos. Anal..

[61] Liu H., Li J., Zhang D. (2023). The effect of thermal times of circulating non-fried roast technique on the formation of (non)volatile compounds in roasted mutton by multi-chromatography techniques and heat transfer analysis. Food Res. Int..

[62] Xiang P., Qiu W., Zheng R. (2021). Dielectric properties of Maillard reaction solutions formed between different amino acids and glucose under microwave heating. Food Bioproc. Tech..

[63] Huang Y., Li H., Huang T. (2014). Lipolysis and lipid oxidation during processing of Chinese traditional smoke-cured bacon. Food Chem..

[64] Wang X., Fan C., Wang X. (2023). Microwave heating and conduction heating pork belly: Influence of heat transfer modes on volatile compounds and aroma attributes. Food Biosci..

[65] Kim J.-S., Lee E.-J., Choi E.H. (2014). Inactivation of *Staphylococcus aureus* on the beef jerky by radio-frequency atmospheric pressure plasma discharge treatment. Innov. Food Sci. Emerg. Technol..

[66] Hunt M.R., Legako J.F., Dinh T.T.N. (2016). Assessment of volatile compounds, neutral and polar lipid fatty acids of four beef muscles from USDA choice and select graded carcasses and their relationships with consumer palatability scores and intramuscular fat content. Meat Sci..

[67] Rejasse B., Lamare S., Legoy M.-D. (2007). Influence of microwave irradiation on enzymatic properties: applications in enzyme chemistry. J. Enzyme Inhib. Med. Chem..

[68] Huang J., Zhao Q., Bu W. (2020). Ultrasound-assisted hydrolysis of lard for free fatty acids catalyzed by combined two lipases in aqueous medium. Bioengineered.

[69] Soares A. de S., Leite Júnior B.R. de C., Tribst A.A.L. (2020). Effect of ultrasound on goat cream hydrolysis by lipase: Evaluation on enzyme, substrate and assisted reaction. LWT Food Sci. Technol..

[70] Jurić S., Jurić M., Siddique M.A.B. (2022). Vegetable oils rich in polyunsaturated fatty acids: nanoencapsulation methods and stability enhancement. Food Rev. Intl..

[71] Shi J., Nian Y., Da D. (2020). Characterization of flavor volatile compounds in sauce spareribs by gas chromatography-mass spectrometry and electronic nose. LWT Food Sci. Technol..

[72] Baggio S.R., Bragagnolo N. (2006). The effect of heat treatment on the cholesterol oxides, cholesterol, total lipid and fatty acid contents of processed meat products. Food Chem..

[73] Tian Z., Zhu Q., Chen Y. (2022). Studies on flavor compounds and free amino acid dynamic characteristics of fermented pork loin ham with a complex starter. Foods.

[74] Wu W., Wang X., Hu P. (2023). Research on flavor characteristics of beef cooked in tomato sour soup by gas chromatography-ion mobility spectrometry and electronic nose. LWT Food Sci. Technol..

[75] Purriños L., Bermúdez R., Franco D. (2011). Development of volatile compounds during the manufacture of dry-cured “Lacón”, a Spanish traditional meat product. J. Food Sci..

[76] Li X., Hu G., Sun X. (2023). The effect of *lactiplantibacillus plantarum* x3–2b bacterial powder on the physicochemical quality and biogenic amines of fermented lamb jerky. Foods.

[77] Li Z., Wang Y., Pan D. (2022). Insight into the relationship between microorganism communities and flavor quality of Chinese dry-cured boneless ham with different quality grades. Food Biosci..

[78] Guo Q., Kong X., Hu C. (2019). Fatty acid content, flavor compounds, and sensory quality of pork loin as affected by dietary supplementation with l-arginine and glutamic acid. J. Food Sci..

[79] Zheng S., Hu Y., Tan L. (2024). Innovative insights into dry fermented sausages flavor: Unraveling the impact of varied *lactobacillus* genera-driven fermentation. Food Biosci..

[80] Xiang J., Wang X., Guo C. (2024). Quality and flavor difference in dry-cured meat treated with low-sodium salts: An emphasis on magnesium. Molecules.

[81] Stahnke L.H. (1994). Aroma components from dried sausages fermented with *Staphylococcus xylosus*. Meat Sci..

[82] Mazubert A., Taylor C., Aubin J. (2014). Key role of temperature monitoring in interpretation of microwave effect on transesterification and esterification reactions for biodiesel production. Bioresour. Technol..

